# The Evolutionary Value of Recombination Is Constrained by Genome Modularity 

**DOI:** 10.1371/journal.pgen.0010051

**Published:** 2005-10-21

**Authors:** Darren P Martin, Eric van der Walt, David Posada, Edward P Rybicki

**Affiliations:** 1 Institute of Infectious Diseases and Molecular Medicine, University of Cape Town, Cape Town, South Africa; 2 Department of Molecular and Cell Biology, University of Cape Town, Cape Town, South Africa; 3 Department of Biochemistry, Genetics and Immunology, University of Vigo, Vigo, Spain; North Carolina State University, United States of America

## Abstract

Genetic recombination is a fundamental evolutionary mechanism promoting biological adaptation. Using engineered recombinants of the small single-stranded DNA plant virus, Maize streak virus (MSV), we experimentally demonstrate that fragments of genetic material only function optimally if they reside within genomes similar to those in which they evolved. The degree of similarity necessary for optimal functionality is correlated with the complexity of intragenomic interaction networks within which genome fragments must function. There is a striking correlation between our experimental results and the types of MSV recombinants that are detectable in nature, indicating that obligatory maintenance of intragenome interaction networks strongly constrains the evolutionary value of recombination for this virus and probably for genomes in general.

## Synopsis

Genetic exchange between organisms, called recombination, occurs in all biological kingdoms and is also common in viruses in which it may threaten the long-term control of important human pathogens such as HIV and influenza. Although recombination can produce advantageous gene combinations, bioinformatic analyses of bacterial genomes have suggested that recombination is not well tolerated when it involves exchanges of genes that interact with a lot of other genes. Using laboratory-constructed recombinants of a small plant virus called MSV, Martin and co-workers provide the first direct experimental evidence that the evolutionary value of exchanging a genome fragment is constrained by the number of ways in which the fragment interacts with the rest of the genome. They note that fitness losses suffered by artificial MSV recombinants increase with decreasing parental relatedness. Furthermore, these losses accurately anticipate the patterns of genetic exchange detectable in natural MSV recombinants, suggesting that they accurately reflect the impact of deleterious selection on natural isolates of the virus.

## Introduction

Genetic recombination may predate the evolution of cellular life [1] and is the basis of ubiquitous biological processes such as DNA repair and sexual reproduction. The combinatorial nature of recombination can provide organisms with vastly more evolutionary options than are available through mutation alone [2–4]. However, kingdom-wide analyses of bacterial recombination [5] and DNA-shuffling studies [6,7] have indicated that the evolutionary value of recombination can vary depending on both the genes and the sub-gene modules transferred. In bacteria, the complexity hypothesis has been proposed to explain an imbalance in detectable informational and operational gene transfers between species [5]. Similarly, the schema hypothesis has been proposed to explain patterns of sequence mosaics observed in DNA-shuffling experiments [7]. Although the complexity hypothesis concerns genes within the context of genomes, the schema hypothesis concerns amino acids within the context of proteins. Both hypotheses are conceptually related and propose that the functionality of sequence fragments in foreign genetic backgrounds is inversely correlated with the complexity of interaction networks within which they must function.

Here we provide experimental support for these hypotheses using the small single stranded plant DNA virus, Maize streak virus (MSV; Geminiviridae, *Mastrevirus*) as a model organism to investigate the effect of genomic recombination on viral fitness.

## Results

### Recombinant Fitness

The MSV genome is approximately 2,690 nucleotides long and contains only three genes and two intergenic regions. We constructed 18 paired reciprocal recombinants (36 genomes in total) from four pairs of MSV isolates sharing genome-wide nucleotide sequence identities of 98%, 95%, 89%, and 78% [8]. Recombinant viruses were constructed in which the three genes and two intergenic regions of MSV were reciprocally exchanged between the four pairs of viruses (Figure 1).

**Figure 1:**
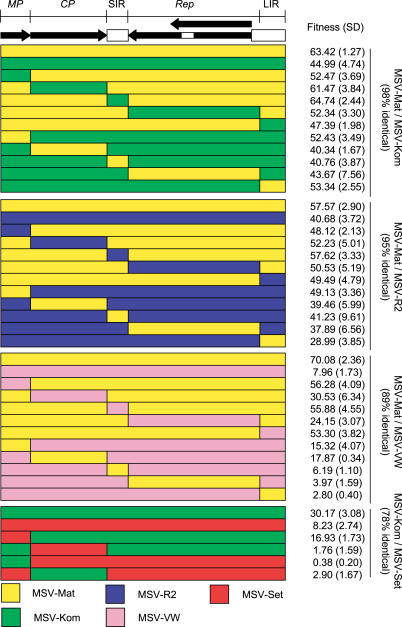
Laboratory Constructed MSV Recombinants and Their Relative Fitness (as Measured by ICLAs) in Maize Five genome regions corresponding to the three MSV genes *(MP, CP,* and *Rep)* and two intergenic regions (LIR and SIR) were reciprocally exchanged between four pairs of MSV isolates (MSV-Mat/MSV-Kom, MSV-Mat/MSV-R2, MSV-Mat/MSV-VW, and MSV-Kom/MSV-Set). Genome-wide sequence identities are indicated. SD, standard deviation.

As a correlate of viral fitness, we determined the induced chlorotic leaf areas (ICLA) of parental and recombinant viruses in infected maize plants [8]. The relationship between symptom severity and fitness is complex for most pathogens. However, MSV populates mesophyll cells within precisely defined chlorotic lesions of infected maize leaves [9] and the chlorotic surface area of an infected leaf is positively correlated with the total amount of viral DNA within the leaf [10,11]. The correlation between MSV pathogenicity and fitness is also evident in the greater geographical distribution and incidence of more pathogenic MSV genotypes relative to less pathogenic genotypes [12].

For each pair of reciprocal recombinants, we defined the recombination tolerance index (Ti) as the average ICLA of the recombinant pair divided by the average ICLA of their parental viruses. If reciprocally exchanged sequence fragments continue to function as well within recombinant genomes as they did in their original genomic backgrounds, we expect that the average ICLA of reciprocal pairs should be identical to that of their parental pairs—i.e., we would expect a Ti = 1.0. Conversely a drop in Ti below 1.0 would indicate that reciprocally exchanged sequences might not function as well in their new genomic backgrounds as they did in their original backgrounds.

In 17 out of 18 recombination experiments, the average ICLA of reciprocal recombinant pairs was lower than that of their parental viruses (i.e., Ti < 1). Values of Ti generally decreased with increasing divergence of exchanged sequences, with a distinct rate of decrease for each genome region exchanged (Figure 2). Given equal degrees of divergence, it seems that the short intergenic region (SIR) and movement protein gene *(MP)* function better in foreign genetic backgrounds than do the replication-associated protein gene *(Rep)*, the coat protein gene *(CP)*, or the long intergenic region (LIR). In other words, the SIR and *MP* appear more modular than the other regions of the genome.

**Figure 2:**
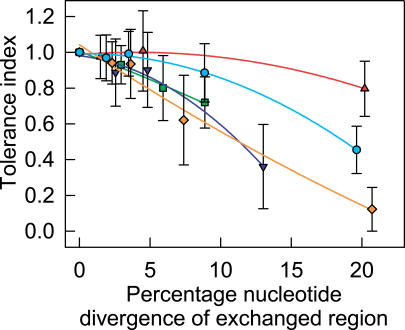
Tolerance of Recombination in MSV Differs According to the Genome Region Involved and the Degree of Divergence between Exchanged Sequences Each plotted point represents a Ti value calculated as the average fitness of a pair of recombinant viruses with reciprocally exchanged *MP* genes (cyan circles), *CP* genes (orange diamonds), *Rep* genes (blue inverted triangles), SIR (red triangles), or LIR (green squares) divided by the average fitness of their parental viruses. Error bars represent the standard deviations of Ti values. Curved lines represent quadratic regressions of Ti values against parental SIR, *MP,* LIR, *CP,* and *Rep* nucleotide sequences.

### Genome Modularity

To better understand these differences in modularity, we examined the network of known direct protein–protein and protein–DNA interactions that occur during an MSV infection of maize (Figure 3) [13–22]. Whereas every other genome component or its expression product participates in multiple specific protein–DNA and/or protein–protein interactions with other virus components, the SIR apparently interacts only with host transcription and DNA replication factors [16]. The only known specific interaction of the *MP* with another virus component is a protein–protein interaction with the *CP* gene [18].

**Figure 3:**
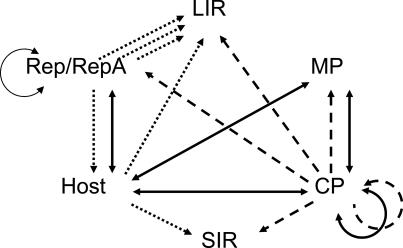
The Network of Known Protein–Protein and Protein–DNA Interactions during a MSV Infection of Maize Rep/RepA indicates the two alternative expression products of the replication-associated protein gene. Solid lines represent specific protein–protein interactions [14,16–18,21,22], dotted lines represent specific protein–DNA interactions [14,16,19–21], and dashed lines indicate CP-DNA interactions of unknown specificity [18–20,22]. For protein–DNA interactions, arrows point from the protein component to the DNA component of the interactions. Rep interacts with the LIR at three distinct sites [14]. CP and Rep form oligomers (solid circular arrows) [14,22]. Although CP must interact with the rest of the genome (including its own gene) during encapsidation, the sequence specificity of these interactions is unknown.

To determine whether the known network of interactions occurring during an MSV infection is anticipated by our Ti data, it was necessary to first extract a relative modularity score for each of the MSV genome regions from the Ti plots. To do this we fitted quadratic equations to the plots (see Figure 2) to estimate similarly tolerable degrees of recombination-induced diversification (RID) in the five genomic regions. Our aim in fitting a line to the Ti plots was to objectively estimate degrees of RID tolerated in the different genome regions at particular Ti values. For example, fitting quadratics to the plots and picking a Ti value of 0.9 (reciprocal recombinants have an average ICLA 90% that of their parents) the corresponding estimates of tolerable recombination induced diversification in the SIR, *MP, CP,* LIR, and *Rep* are 15.3%, 8.3%, 2.7%, 3.3%, and 3.8% respectively. To avoid any biases due to “cherry picking” the Ti values we used to compare the different genome regions, we examined estimated recombination tolerances over the range of Ti values between 0.99 and 0.7. There is good correlation between the number of known MSV intergenome component protein–protein and protein–DNA interactions for different genome regions (SIR = 1, *MP* = 2, *CP* = 5, LIR = 4, and *Rep* = 4) and recombination tolerance estimated for these same regions over the entire Ti range between 0.94 and 0.7 (Pearson's *R* ^2^ > 0.87, *p* < 0.05, Spearman's rho corrected for ties = −0.975, *p* = 0.051).

### Analysis of Natural Recombinants

To investigate whether experimentally determined Ti values provide any insights into real processes that influence the survival of recombinants in nature, we examined all available *Mastrevirus* sequences in GenBank for evidence of recombination. In each of the five genomic regions (LIR, *MP, CP,* SIR, and *Rep*), we identified evidence for unique recombination events involving MSV isolates or the closely related African streak viruses. We only retained evidence of recombination events detectable in genomes with proven viability (as determined by the existence of infectious clones of these genomes). For each recombination event involving two identifiable parental sequences and comprising two easily identifiable breakpoints both unambiguously within one of the five defined genomic regions, we used the identified parental sequences to infer the number of nucleotide differences between the transferred sequence and the sequence it replaced (Table 1). The greatest number of nucleotide changes observed in a single recombination event in each of the five genomic regions provides a gross estimate of the maximum parental divergence tolerated in nature. The set of values thus determined (SIR = 15.0%, *MP* = 7.7%, *CP* = 3.2%, LIR = 5.4%, and *Rep* = 3.9%) was significantly correlated with equivalent sets of values derived from the quadratic regressions shown in Figure 2 (Figure 4; Pearson's *R* ^2^ > 0.96, *p* < 0.01, Spearman's rho = 0.9, *p* = 0.037 over the Ti range 0.95–0.7).

**Table 1:**
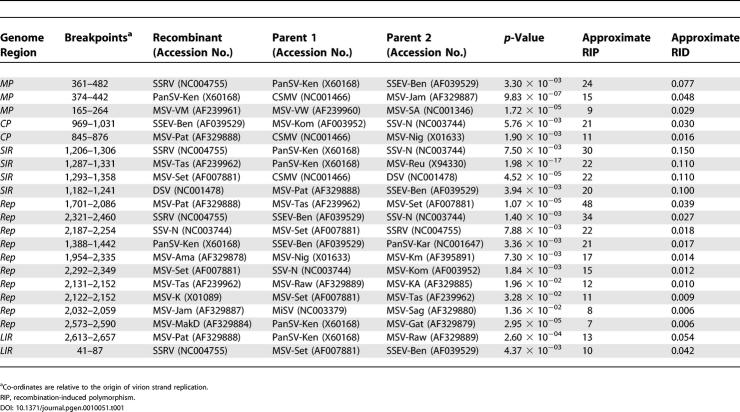
Evidence of Recombination within Five Defined Regions of Publicly Available African Streak Virus Genome Sequences

**Figure 4:**
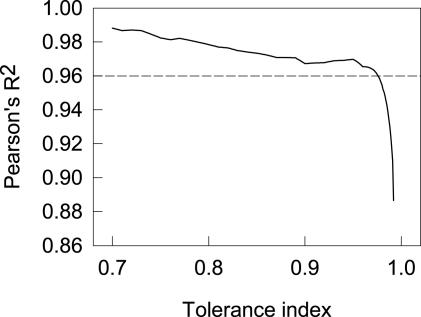
The Experimentally Determined Relative Recombination Tolerance of Five MSV Genomic Regions Is Highly Correlated with Values Inferred from Nature We used the quadratic regressions presented in Figure 2 to derive experimental estimates of similarly tolerable degrees of RID in the five genomic regions over a range of Ti values between 0.99 and 0.70 (290 values at 0.01 intervals). Using a range of Ti values avoids any biases that might occur due to inadvertently choosing particularly poor/favourable Ti values for estimating similarly tolerable degrees of RID from the experimental data. For example, the set of similarly tolerable degrees of RID calculated when Ti = 0.9 is 15.3%, 8.3%, 2.7%, 3.3%, and 3.8% for the SIR, *MP* gene, *CP* gene, LIR, and *Rep* gene, respectively. Each of the 290 sets of values thus determined was linearly regressed against the set of values for the maximum tolerable RID inferred for the same five regions from an examination of natural recombinants (15.0%, 7.7%, 3.2%, 5.4%, and 3.9%, for SIR, *MP, CP,* LIR, and *Rep,* respectively). *R* ^2^ values determined for these 290 regressions are plotted against their corresponding Ti values (solid line). The correlation is significant (broken line = *R* ^2^ value corresponding to a *p*-value < 0.01) over the Ti range 0.95–0.7 (Pearson's *R* ^2^ > 0.96, *p* < 0.01; Spearman's rho = 0.9, *p* = 0.037).

## Discussion

By demonstrating a negative correlation between the relative modularity of defined genomic regions and the complexity of interactions in which they are involved we have provided experimental support for the complexity hypothesis [5]. This hypothesis was proposed to explain the disparity in informational (those involved in transcription, translation, and related processes) and operational (those involved in housekeeping) gene transfer rates in bacteria. It states that because informational genes are generally involved in more complex interactions than operational genes, they are less likely to continue functioning well after horizontal transfer.

The progressive decrease in tolerance of recombination with increasing divergence of exchanged sequences observed in Figure 2 has strong parallels with parental sequence imbalances observed in “family shuffling” variants of DNA-shuffling experiments [2]. The functional genes produced by shuffling three or more distinct sequences (i.e., 60%–85% identical) are usually derived predominantly from either one sequence or combinations of the most similar sequences [2,6,23,24]. The schema hypothesis proposes that these imbalances are due to the probability of recombinant protein-fold disruption increasing with increasing divergence of parental sequences [6,7]. We shuffled entire intergenic regions and genes, and therefore the effect we observed cannot be explained directly in terms of the schema hypothesis. We have, however, provided empirical evidence for a whole-genome analogue of this hypothesis: The probability of the normal network of intragenome interactions being disrupted by recombination increases with increasing divergence of the exchanged fragments.

The successful inheritance of genomic fragments through recombination is expected to depend on the maintenance of important intragenome interactions. After all, the exchange of a genome fragment could be seen as a simultaneous introduction of multiple mutations. Negative or purifying selection should remove those recombinants that break the epistatic interactions that define the architecture of a particular genome, whereas genetic drift might permit the survival and spread of “neutral” recombinants. Alternatively, positive selection should favour the spread of rare recombinants with improved genomic interactions. The genomic interactions in the (natural) parental viruses used in these experiments have most likely been optimised through selection over long evolutionary periods. None of the recombinants generated from these viruses was more fit than the fitter of its parents, which is expected if negative selection is the dominant force that now maintains the integrity of these genomes.

The relative degree of modularity that we demonstrated experimentally for each genome component appears to be reflected in the recombination events detected within the same regions in natural viruses represented in GenBank. This correlation is surprising because the natural recombinants—unlike the recombinants we constructed in the laboratory—involve exchanges of fragments of genes or intergenic regions. Such exchanges may disrupt intraprotein or intra-intergenic region interactions as well as interaction networks amongst whole genes and intergenic regions. Survival of the natural viruses with detectable recombination events in coding regions presumably depended on their inheritance of sequences that did not overly disrupt either intraprotein or intergene/intergenic region interaction networks; survival of natural intergenic region recombinants and those we generated in the laboratory would have been subject only to the latter constraint. The correlation between our experimental results and the inferred natural recombinants may indicate that maintenance of intergenome component interactions is the principal determinant of recombination tolerance (at least for MSV and closely related viruses). Alternatively, a requirement for the preservation of both intergenome component, and intragene, interaction networks has a net effect that is difficult to distinguish from either constraint operating alone.

We have provided experimental support for the complexity hypothesis by demonstrating a relationship between the relative modularity of defined genomic regions and the complexity of interactions in which they are involved. The striking correlation between our experimental results and the types of recombination observed in nature lends credence to the notion that these detectable modularity differences are evolutionarily relevant. Our results also suggest that the degree of similarity between an inherited sequence and the sequence it replaces is an important additional determinant of recombinant fitness. Whereas recombination can substantially increase the evolutionary options of an organism, the obligatory maintenance of co-evolved interaction networks may severely restrict its evolutionary value.

## Materials and Methods

### Recombinant construction.

We have previously described the construction and symptom analysis of 15 reciprocal recombinant pairs for the MSV isolates MSV-Mat, MSV-Kom, MSV-R2, and MSV-VW [8]. PCR mutagenesis was used to introduce NcoI restriction sites immediately upstream of the *CP* gene start codons of the MSV isolates MSV-Set and MSV-Kom to obtain SNco and KNco, respectively. Reciprocal *MP* gene recombinants were obtained by exchanging BamHI-NcoI restriction fragments containing the entire *MP* between MSV-SNco and MSV-KNco. Reciprocal *CP* recombinants were obtained by exchanging NcoI-NcoI restriction fragments containing the entire *CP* between MSV-SNco and MSV-KNco. Agroinfectious partially dimeric clones of recombinant viruses were constructed as previously described [10].

### Fitness testing.

The fitness of recombinant and parental viruses as indicated by ICLA was determined in maize by agroinoculation of 3-d-old cv. Jubilee seedlings with image analysis quantification of ensuing disease symptoms [8]. An ICLA score for each virus was determined by averaging the percentage chlorotic areas determined on leaves two through six for between three and 14 symptomatic plants in each of three to seven replicated agroinoculation experiments.

### Recombination detection.

All available *Mastrevirus* sequences were obtained from GenBank and aligned using POA [25] with gap open and gap extension penalties of 12 and 6, respectively. Identification of potential recombinant and parental sequences, and localisation of possible recombination breakpoints was carried out using the RDP [26], Geneconv [27], RecScan [28], Maximum Chi Square [29], Chimaera [30], and SisterScan [31] methods as implemented in RDP2 [30]. The analysis was performed with default settings for the detection methods, a Bonferroni corrected *p*-value cutoff of 0.05, and a requirement that any potential event be detectable by two or more methods.

## Supporting Information

### Accession Numbers

The GenBank (http://www.ncbi.nlm.nih.gov/Genbank) accession numbers for the genome sequences are CSMV (NC001466), DSV (NC001478), MiSV (NC003379), MSV-Ama (AF329878), MSV-Gat (AF329879), MSV-Jam (AF329887), MSV-K (X01089), MSV-KA (AF329885), MSV-Km (AF395891), MSV-Kom (AF003952), MSV-MakD (AF329884), MSV-Nig (X01633), MSV-Pat (AF329888), MSV-Raw (AF329889), MSV-Reu (X94330), MSV-SA (NC001346), MSV-Sag (AF329880), MSV-Set (AF007881), MSV-Tas (AF239962), MSV-VM (AF239961), MSV-VW (AF239960), PanSV-Kar (NC001647), PanSV-Ken (X60168), SSEV-Ben (AF039529), SSRV (NC004755), and SSV-N (NC003744).

## Abbreviations

ICLA: induced chlorotic leaf area
LIR: long interegenic region
MSV: Maize streak virus
RID: recombination-induced diversification
SIR: short intergenic region
Ti: tolerance index

## References

[pgen-0010051-b01] Lehman NA (2003). A case for the extreme antiquity of recombination. J Mol Evol.

[pgen-0010051-b02] Crameri A, Raillard SA, Bermudez E, Stemmer WP (1998). DNA shuffling of a family of genes from diverse species accelerates directed evolution. Nature.

[pgen-0010051-b03] Drummond DA, Silberg JJ, Meyer MM, Wilke CO, Arnold FH (2005). On the conservative nature of intragenic recombination. Proc Natl Acad Sci U S A.

[pgen-0010051-b04] Stemmer WPC (1994). Rapid evolution of a protein in vitro by DNA shuffling. Nature.

[pgen-0010051-b05] Jain R, Rivera MC, Lake JA (1999). Horizontal gene transfer among genomes: The complexity hypothesis. Proc Natl Acad Sci U S A.

[pgen-0010051-b06] Meyer MM, Silberg JJ, Voigt CA, Endelman JB, Mayo SL (2003). Library analysis of SCHEMA-guided protein recombination. Prot Sci.

[pgen-0010051-b07] Voigt CA, Martinez C, Wang ZG, Mayo SL, Arnold FH (2002). Protein building blocks preserved by recombination. Nat Struct Biol.

[pgen-0010051-b08] Martin DP, Rybicki EP (2002). Investigation of Maize streak virus pathogenicity determinants using chimaeric genomes. Virology.

[pgen-0010051-b09] Lucy AP, Boulton MI, Davies JW, Maule AJ (1996). Tissue specificity of *Zea mays* infection by maize streak virus. Mol Plant Microbe Interact.

[pgen-0010051-b10] Schnippenkoetter WH, Martin DP, Willment JA, Rybicki EP (2001). Forced recombination between distinct strains of Maize streak virus. J Gen Virol.

[pgen-0010051-b11] Shepherd DN, Martin DP, McGivern DR, Boulton MI, Thomson JA (2005). A three-nucleotide mutation altering the Maize streak virus Rep pRBR-interaction motif reduces symptom severity in maize and partially reverts at high frequency without restoring pRBR-Rep binding. J Gen Virol.

[pgen-0010051-b12] Martin DP, Willment JA, Billharz R, Velders R, Odhiambo B (2001). Sequence diversity and virulence in *Zea mays* of Maize streak virus isolates. Virology.

[pgen-0010051-b13] Boulton MI (2002). Functions and interactions of mastrevirus gene products. Phys Mol Plant Path.

[pgen-0010051-b14] Castellano MM, Sanz-Burgos AP, Gutierrez C (1999). Initiation of DNA replication in a eukaryotic rolling-circle replicon: Identification of multiple DNA-protein complexes at the geminivirus origin. J Mol Biol.

[pgen-0010051-b15] Dickinson VJ, Halder J, Woolston CJ (1996). The product of maize streak virus ORF V1 is associated with secondary plasmodesmata and is first detected with the onset of viral lesions. Virology.

[pgen-0010051-b16] Donson J, Morris-Krsinich BA, Mullineaux PM, Boulton MI, Davies JW (1984). A putative primer for second-strand DNA synthesis of maize streak virus is virion-associated. EMBO J.

[pgen-0010051-b17] Horvath GV, Pettko-Szandtner A, Nikovics K, Bilgin M, Boulton M (1998). Prediction of functional regions of the maize streak virus replication-associated proteins by protein-protein interaction analysis. Plant Mol Biol.

[pgen-0010051-b18] Liu H, Boulton MI, Oparka KJ, Davies JW (2001). Interaction of the movement and coat proteins of Maize streak virus: Implications for the transport of viral DNA. J Gen Virol.

[pgen-0010051-b19] Liu H, Boulton MI, Thomas CL, Prior DA, Oparka KJ (1999). Maize streak virus coat protein is karyophyllic and facilitates nuclear transport of viral DNA. Mol Plant Microbe Interact.

[pgen-0010051-b20] Liu H, Boulton MI, Davies JW (1997). Maize streak virus coat protein binds single- and double-stranded DNA in vitro. J Gen Virol.

[pgen-0010051-b21] Nikovics K, Simidjieva J, Peres A, Ayaydin F, Pasternak T (2001). Cell-cycle, phase-specific activation of Maize streak virus promoters. Mol Plant Microbe Interact.

[pgen-0010051-b22] Zhang W, Olson NH, Baker TS, Faulkner L, Agbandje-McKenna M (2001). Structure of the Maize streak virus geminate particle. Virology.

[pgen-0010051-b23] Aharoni A, Gaidukov L, Yagur S, Toker L, Silman I (2004). Directed evolution of mammalian paraoxonases PON1 and PON3 for bacterial expression and catalytic specialization. Proc Natl Acad Sci U S A.

[pgen-0010051-b24] Joern JM, Meinhold P, Arnold FH (2002). Analysis of shuffled gene libraries. J Mol Biol.

[pgen-0010051-b25] Lee C, Grasso C, Sharlow MF (2002). Multiple sequence alignment using partial order graphs. Bioinformatics.

[pgen-0010051-b26] Martin D, Rybicki E (2000). RDP: Detection of recombination amongst aligned sequences. Bioinformatics.

[pgen-0010051-b27] Padidam M, Sawyer S, Fauquet CM (1999). Possible emergence of new geminiviruses by frequent recombination. Virology.

[pgen-0010051-b28] Martin DP, Posada D, Crandall KA, Williamson CA (2005). A modified bootscan algorithm for automated identification of recombinant sequences and recombination breakpoints. AIDS Hum Retro.

[pgen-0010051-b29] Smith JM (1992). Analyzing the mosaic structure of genes. J Mol Evol.

[pgen-0010051-b30] Martin DP, Williamson C, Posada D (2005). RDP2: Recombination detection and analysis from sequence alignments. Bioinformatics.

[pgen-0010051-b31] Gibbs MJ, Armstrong JS, Gibbs AJ (2000). Sister-scanning: a Monte Carlo procedure for assessing signals in recombinant sequences. Bioinformatics.

